# Effects of the Web Behavior Change Program for Activity and Multimodal Pain Rehabilitation: Randomized Controlled Trial

**DOI:** 10.2196/jmir.5634

**Published:** 2016-10-05

**Authors:** Catharina A Nordin, Peter Michaelson, Gunvor Gard, Margareta K Eriksson

**Affiliations:** ^1^ Department of Health Science Division of Health and Rehabilitation Luleå University of Technology Luleå Sweden; ^2^ Department of Primary Health Care Norrbotten County Council Piteå Sweden; ^3^ Department of Public Health Norrbotten County Council Luleå Sweden

**Keywords:** coping behavior, pain, patient compliance, patient satisfaction, rehabilitation, self-efficacy, Web-based intervention

## Abstract

**Background:**

Web-based interventions with a focus on behavior change have been used for pain management, but studies of Web-based interventions integrated in clinical practice are lacking. To emphasize the development of cognitive skills and behavior, and to increase activity and self-care in rehabilitation, the Web Behavior Change Program for Activity (Web-BCPA) was developed and added to multimodal pain rehabilitation (MMR).

**Objective:**

The objective of our study was to evaluate the effects of MMR in combination with the Web-BCPA compared with MMR among persons with persistent musculoskeletal pain in primary health care on pain intensity, self-efficacy, and copying, as part of a larger collection of data. Web-BCPA adherence and feasibility, as well as treatment satisfaction, were also investigated.

**Methods:**

A total of 109 participants, mean age 43 (SD 11) years, with persistent pain in the back, neck, shoulder, and/or generalized pain were recruited to a randomized controlled trial with two intervention arms: (1) MMR+WEB (n=60) and (2) MMR (n=49). Participants in the MMR+WEB group self-guided through the eight modules of the Web-BCPA: pain, activity, behavior, stress and thoughts, sleep and negative thoughts, communication and self-esteem, solutions, and maintenance and progress. Data were collected with a questionnaire at baseline and at 4 and 12 months. Outcome measures were pain intensity (Visual Analog Scale), self-efficacy to control pain and to control other symptoms (Arthritis Self-Efficacy Scale), general self-efficacy (General Self-Efficacy Scale), and coping (two-item Coping Strategies Questionnaire; CSQ). Web-BCPA adherence was measured as minutes spent in the program. Satisfaction and Web-BCPA feasibility were assessed by a set of items.

**Results:**

Of 109 participants, 99 received the allocated intervention (MMR+WEB: n=55; MMR: n=44); 88 of 99 (82%) completed the baseline and follow-up questionnaires. Intention-to-treat analyses were performed with a sample size of 99. The MMR+WEB intervention was effective over time (time*group) compared to MMR for the two-item CSQ catastrophizing subscale (*P*=.003), with an effect size of 0.61 (Cohen *d*) at 12 months. There were no significant between-group differences over time (time*group) regarding pain intensity, self-efficacy (pain, other symptoms, and general), or regarding six subscales of the two-item CSQ. Improvements over time (time) for the whole study group were found regarding mean (*P*<.001) and maximum (*P*=.002) pain intensity. The mean time spent in the Web-based program was 304 minutes (range 0-1142). Participants rated the items of Web-BCPA feasibility between 68/100 and 90/100. Participants in the MMR+WEB group were more satisfied with their MMR at 4 months (*P*<.001) and at 12 months (*P*=.003).

**Conclusions:**

Adding a self-guided Web-based intervention with a focus on behavioral change for activity to MMR can reduce catastrophizing and increase satisfaction with MMR. Patients in MMR may need more supportive coaching to increase adherence in the Web-BCPA to find it valuable.

**ClinicalTrial:**

Clinicaltrials.gov NCT01475591; https://clinicaltrials.gov/ct2/show/NCT01475591 (Archived by WebCite at http://www.webcitation.org/6kUnt7VQh)

## Introduction

Internet-based medicine or eHealth is under continuous development and considered necessary to provide cost-effective and equal health care [1]. The eHealth definition comprises Internet technology and a commitment to improve the quality of and access to health care by the use of information and communication technology, as well as empowering the individual and increasing participation [2]. Web-based interventions for pain management have been developed and promising treatment effects regarding pain and physical and psychological functioning have been reported [3-5].

Approximately 20% of the adult Swedish and European population suffers from persistent musculoskeletal pain with duration of at least 3 months or recurrent episodes of pain [6,7]. For the individual, persistent musculoskeletal pain is reported to have an impact on the individual’s quality of life [6,8] and imposes high societal costs with large health care consumption, work absenteeism, and sick leave [9,10]. The influence of psychosocial factors [11-13] and reported comorbidity [14] in persistent musculoskeletal pain entail a biopsychosocial and holistic approach to treatment, such as multimodal rehabilitation (MMR) [7,15-17]. The treatment content in MMR can vary, but includes at least a physical (body exercises) and a psychosocial (psychological, social, or occupational) component [15,18], given by health care professionals of different occupations [17,19]. MMR includes a cognitive behavioral approach to help the individual to understand how cognition and behavior can affect the pain experience and to provide tools for self-care [18]. The individual’s active participation in rehabilitation planning and decision making, including setting goals with a focus on participation in daily life and work, have been emphasized [7,16,17,20]. There is evidence for MMR when compared to standard treatment regarding reduced pain intensity and improved functioning [15,19,21], as well as reduced social costs with fewer days of sick leave [22]. However, some reports have demonstrated ambiguous and mixed results [15,23]. The treatment effects of MMR have been associated with the individual’s changes in beliefs and coping [24]. Self-efficacy has been found to mediate a positive treatment outcome [25-27] and to be important in the use of more active coping strategies and self-management [28,29]. In contrast, catastrophizing beliefs have a negative impact on treatment effects [24,26,30]. Although MMR is the recommended treatment for persistent pain, there are reasons for further improvements within treatment content for persistent pain.

In the County Council of Norrbotten, Sweden, the development of eHealth care is a strategy to overcome the regional distance between health care providers and citizens. In order to propose an eHealth solution for a biopsychosocial treatment of persistent musculoskeletal pain, the Web-based Behavior Change Program for Activity (Web-BCPA) was developed. The Web-BCPA is a modified version of an existing Web-based program “To Manage Pain” provided by Livanda (a Swedish supplier of Internet-based medicine) [31]. To Manage Pain is based on behavioral theory literature and face-to-face cognitive behavioral therapy [32-34], and was developed by psychologists of the Livanda company [31]. In cooperation with the founders of Livanda, To Manage Pain was revised into the Web-BCPA program with the aim to target patients in an early stage of persistent pain. The Web-BCPA aimed to increase participants’ physical and cognitive activity in the rehabilitation. The Web-BCPA focuses on increasing cognitive activities, such as learning, problem solving, communication, and making decisions, to help the participants develop new skills and behavior, as well as maintain and generalize behavior changes in life. Further, the Web-BCPA content was designed to encourage activity in everyday life and work, as well as physical activity and self-care.

At the time of this study, there were no interventions combining MMR with a self-guided Web-based intervention for pain management and behavior change. Most studies on Web-based interventions had participants recruited from waiting lists and/or advertising, which indicated that further research needed to focus on integrating Web-based interventions in clinical practice [3,4,35], including evaluations of treatment satisfaction and feasibility [4]. In addition, few studies have evaluated self-guided Web-based interventions with no therapist support [36-39]. We chose to perform our study in the primary health care setting because earlier research on MMR focused on in-patient intervention and there was a lack of studies performed in outpatient rehabilitation of persons with persistent musculoskeletal pain [40].This study is part of a larger collection of data with the main objective to evaluate work ability. Here, we focus on reporting the results of other outcomes in relation to pain to evaluate the Web-BCPA program. The objective of this study was to evaluate the effects of MMR in combination with the Web-BCPA compared to MMR among persons with persistent musculoskeletal pain in primary health care regarding pain intensity, self-efficacy, and coping. The study also aimed to investigate Web-BCPA adherence and feasibility, as well as treatment satisfaction.

## Methods

### Study Design

The study was a 12-month randomized controlled trial (RCT) with two intervention arms: (1) MMR and the Web-BCPA (MMR+WEB) and (2) MMR with follow-ups at 4 and 12 months. The consecutive recruitment and data collection started in October 2011 and ended in May 2015. The protocol was registered in the clinical trial registry of the US National Institutes of Health (NCT01475591), and approved by the Regional Ethical Review Board of Umeå University, Sweden (Umu dnr 2011-383-31M). This study is part of a larger collection of data and focuses on evaluating Web-BCPA adherence and feasibility, as well as outcomes of self-efficacy, pain intensity, and coping strategies.

### Participants

Participants were patients eligible for MMR at health care centers in Norrbotten county, northern Sweden. The inclusion criteria were (1) age between 18 and 63 years; (2) persistent musculoskeletal pain with a duration of at least 3 months in back, neck, shoulder, and/or generalized pain; (3) Örebro Musculoskeletal Pain Screening Questionnaire (ÖMPSQ) score ≥90, screening for psychosocial factors that indicates an estimated risk for long-lasting pain conditions and future disability [12]; (4) work ability of at least 25%; (5) familiar with written and spoken Swedish; and (6) access to a computer and the Internet. Exclusion criteria were reduced cognitive ability (dementia, brain injury), current abuse of alcohol or drugs, in need of other health care (eg, advanced medical investigation, cancer treatment, terminal care), and/or pregnancy.

### Procedure

We invited 23 primary health care centers in Norrbotten that were certified for MMR to participate in the study. Management and health care staff were briefed and the rehabilitation coordinator (nurse, occupational therapist, or physiotherapist assigned to support a patient in rehabilitation planning) was trained to assist in the recruitment and data collection as well as introducing the participants to self-guide the Web-BCPA.

In all, 17 health care centers actively participated in the study. The rehabilitation coordinator at each health care center selected the participants according to inclusion and exclusion criteria. When patients were considered eligible for study participation, oral and written information about the study was provided and the patient was asked about participation. Once informed consent was obtained, the participants filled in the baseline questionnaire and were then randomly allocated to either the MMR+WEB group or the MMR group by numbered opaque envelopes. An independent statistician provided the allocation sequences by computer-generated random number sequences for each health care center and stratified by sex before inclusion of participants.

Participants in both intervention groups started MMR treatment according to their rehabilitation plan. Participants allocated to the MMR+WEB group were assisted by the rehabilitation coordinator to form their username and to self-select a password to log in to the Web-BCPA. They were instructed about the general setup of the Web-based intervention and informed that the rehabilitation coordinator was available for support. In addition, participants were informed that the time spent on the Web-BCPA was to be monitored and that participants who did not log in to the program would be contacted by the rehabilitation coordinator.

Participants in both study groups were followed up at 4 and 12 months. On both occasions, the participants met with the rehabilitation coordinator at the health care center and filled in a questionnaire. In addition, the participants were asked for consent to review their patient records for data on number of treatments and sick-leave days.

### Interventions

#### Multimodal Rehabilitation

The MMR was characterized by synchronized treatments based on a biopsychosocial perspective of pain and with the patient in focus. The MMR included treatments from at least three health care professionals from different occupations (eg, nurse, occupational therapist, physician, physiotherapist, psychologist, or psychosocial counselor). The health care professionals worked according to the cognitive behavioral approach for behavior change toward activity and participatory goals. In addition, the participants and the health care professionals were supported by a rehabilitation coordinator in the planning of the rehabilitation and in communication with the Swedish Social Insurance Agency (SSIA). The patient and the health care professionals met at team conference meetings to draw up an individualized rehabilitation plan, which included identification of the patient’s resources and restrictions, formulation of goals, planning of treatments, as well as dates for follow-up. The plan was documented by a standard form in the patient record and printed out for the participants. The participants had the opportunity to invite significant others (a relative, an employer, an administrator from the SSIA or the Employment Service) to cooperate in the rehabilitation planning. Mutual decision making and a patient’s active participation in MMR treatments and planning were in focus [16,17].

The minimum number of treatments in MMR was specified as two to three times a week for six to eight weeks, including home exercises. The treatments were individual and/or in group sessions. In MMR physical activity (individualized exercise program, warm-water exercise, Basic Body Awareness Therapy), acupuncture, transcutaneous electric nerve stimulation, and manual therapy could be given by physiotherapists. Ergonomics, activity planning, and functional training were provided by occupational therapists. Psychologists and psychosocial counselors were responsible for counseling treatment. Counseling could also be provided by other health care professionals (nurse, occupational therapist, or physiotherapist) trained in cognitive behavioral therapy. The physicians prescribed pharmacological treatment, wrote medical certificates, and made referrals. Patient education, relaxation, mindfulness, and testing disability aids were carried out by health care professionals of various occupations. The MMR treatment period was adjusted according to the patient’s needs and progress. The health care centers were responsible for a patient’s medical rehabilitation to progress in health, but not principally in charge of the work rehabilitation.

#### The Web Behavior Change Program for Activity

The Web-BCPA was administrated via the Livanda website, and was exclusive for this study. Only study participants had access to the Web-BCPA, not other Livanda customers. The participants self-guided through the Web-BCPA, without therapist guidance, and had the freedom to choose from the program content. They had access to the Web-based intervention in their own environment 24/7 for 16 weeks. Without participants’ active work in the Web-BCPA for 20 minutes, they were automatically logged out. At the first log-in, the Web-BCPA contained an overall introduction to cognitive behavioral therapy principles, information of the content and format of the entire program, as well as general advice on how to work in the Web-BCPA (eg, start with reading the texts and then spend time on the assignments). The Web-BCPA consisted of eight modules: (1) pain, (2) activity, (3) behavior, (4) stress and thoughts, (5) sleep and negative thoughts, (6) communication and self-esteem, (7) solutions, and (8) maintenance and progress. They were delivered to the participant one module per week during the first eight weeks. The modules contained information, assignments, and exercises, assimilated via educational texts, videos, and writing tasks. Each module contained 10 to 15 shorter Web pages of information and 10 to 15 pages of assignments and exercises (Table 1). Further, the assignments were interactive and included self-tests and self-developed action plans aimed at self-analyzing one’s resources and restrictions, setting goals and estimating goal achievement, planning activities, and following up results. Help texts with specific how-to instructions, as well as examples of goals and activities, were available to all assignments. Self-developed action plans included assignments on life goals and values, activity scheduling, and planning behavior change. Exercises included relaxation and Basic Body Awareness Therapy exercises, for example, with a duration of 10 to 30 minutes per session. In addition, the participant could choose any physical activity as part of the planning activity assignment. Assignments and exercises were constructed as a progression in cognitive skill building with each module. The participants chose how to use the Web-BCPA freely, except for a well-being test that was mandatory to fill in to get access to modules 2 to 8. The well-being test measured harmony (in contrast to anxiety), energy level, optimism (in contrast to depression), and decisiveness. Data from the well-being test and the assignments were saved as summaries, which the participants could review to monitor progress. All texts and assignments could be printed out. If participants’ chose, complementary well-being recommendations were sent to the participant’s email box each week. In addition, the program included a CD with relaxation exercises, which was sent to their home address.

### Outcome Measures

#### Web Behavior Change Program for Activity Adherence

Web-BCPA adherence was assessed as minutes spent in each module, which was obtained from the administrative system of Livanda. Total time was calculated.

#### Web Behavior Change Program for Activity Feasibility and Treatment Satisfaction

Web-BCPA feasibility was measured at 4 months using a set of items constructed for the purpose of this study. The eight items were:

1. It was easy to use the program

2. It was easy to log in to the program

3. Except for the first introduction, I have self-guided in the program

4. It was easy to comprehend the program

5. The graphical design was...

6. The texts have been of good use

7. The exercises have been of good use

8. The videos have been of good use

The ranking was made on a numeric scale from zero (disagree) to 100 (totally agree). The score for item 5 was zero (not at all appealing) to 100 (appealing).

Participants’ satisfaction with the Web-BPCA was measured at 4 months with three items: (1) I am satisfied with my own efforts in the Web-based intervention, (2) I am satisfied with the administrative support in the Web-based intervention from the rehabilitation coordinator, and (3) I could recommend the Web-based intervention to others in a similar situation as mine.

In addition, participants’ satisfaction with the MMR was assessed at 4 and 12 months using two items: (1) I am satisfied with my multimodal rehabilitation, and (2) I am satisfied with my own efforts in my multimodal rehabilitation. The ratings were on a numeric scale from zero (disagree) to 100 (totally agree).

#### Patient Records Data

Data on MMR treatment, health care consumption, and sick leave were collected from the participant’s patient records.

#### Pain Intensity

Pain intensity was measured by the 100-mm Visual Analog Scale (VAS) with zero indicating no pain or discomfort and 100 indicating unbearable pain or discomfort [41]. The participants assessed their mean, minimum, and maximum pain for the last seven days [42]. The VAS has good reliability and is well established to assess musculoskeletal pain [43].

#### Self-Efficacy

##### Arthritis Self-Efficacy Scale

The certainty to have the capacity to perform a task in relation to pain was measured with two subscales of the Arthritis Self-Efficacy Scale (ASES). The “self-efficacy to control pain” subscale (ASES pain) consisted of five items and the “self-efficacy to control other symptoms” subscale (ASES other symptoms) had six items. The items were scored on a scale from 10 (very uncertain) to 100 (very certain), with a mean score for each subscale computed [39]. Both the original ASES and the Swedish version have been tested for reliability (alpha range .8 to .9) and validity [44-46].

**Table 1:** Content of the Web Behavior Change Program for Activity (Web-BCPA).

Module	Educational texts	Assignments and exercises
1. Pain	Pain mechanism—anatomy and physiology	Life goals and values—health
	Persistent pain	Activity scheduling
	Pain in the neck, back, and shoulder	
2. Activity	Pain mechanism—thoughts, interpretation, behavior	Well-being test
	Pain and physical activity	Life goals and values—work and leisure
	Life balance	Daily exercise level test
	Ergonomics in everyday life	Short exercise program
	Resting positions	Relaxation—breathing exercises
		Basic Body Awareness Therapy exercises
3. Behavior	Pain and learning behavior	Well-being test
	Pacing	Life goals and values—close relationships, family, social relationships, and personal development
	An active sick-leave	Planning activity
		Planning behavior change
		Body scan-applied relaxation
		Basic Body Awareness Therapy exercises
4. Stress and thoughts	Accepting thoughts	Well-being test
	Stress and stress management	Planning behavior change
		Stress test
		Body scan—conditioned relaxation
		Basic Body Awareness Therapy exercises
5. Sleep and negative thoughts	Negative and automatic thoughts	Well-being test
	Sleep, sleep hygiene, and sleep disorders	Challenging negative automatic thinking styles
		Sleep test
		Body scan—conditioned relaxation
		Basic Body Awareness Therapy exercises
6. Communication and self-esteem	Communication skills	Well-being test
	Conflict resolution methods	Effective communication training
	Self-esteem and self-confidence	Setting limits
	Participation in health care	Dealing with difficult emotions
		Planning behavior change
		Basic Body Awareness Therapy exercises
7.Solutions	Problem-solving methods in relationships	Well-being test
	Problem-solving traps	Problem-solving practices
		Planning behavior change
		Basic Body Awareness Therapy exercises
8. Maintenance and progress	Setbacks and relapses prevention	Well-being test
	Maintenance	Planning behavior change
		Maintenance plan and strategies
		Basic Body Awareness Therapy exercises

##### General Self-Efficacy Scale

A more general aspect of self-efficacy was assessed by the General Self-Efficacy Scale (GSE), which measures an individual’s beliefs in one’s ability to respond to novel or difficult situations and to deal with associated obstacles or setbacks. The GSE contained 10 items, which were rated on a four-point Likert scale: 1 (not at all true/strongly disagree), 2 (hardly true/partly disagree), 3 (moderately true/partly agree), and 4 (exactly true/strongly agree). The ratings were summarized and divided by 10, resulting in a total score ranging from 1 to 4 [47-49]. The GSE was found consistent (alpha range .7 to .9) in several populations in European countries [50], and the Swedish version has been validated [49].

#### Coping

Coping strategies were assessed using the two-item Coping Strategies Questionnaire (CSQ), a shorter version of the original CSQ. The two-item CSQ consists of seven subscales, each represented by two items [51]. The subscales represent a coping strategy: diverting attention, reinterpreting pain sensations, catastrophizing, ignoring sensations, praying or hoping, coping self-statements, and increased behavioral activities. The items were scored on a Likert scale from zero (never do that) to 6 (always do that), and a mean score of the two items for each subscale was calculated. A higher score is related to improvement of coping strategies, except for the catastrophizing subscale in which a lower score indicates improvement. Each of the CSQ two-item subscales has shown strong association to the parent subscale [51]. A Swedish version of the two-item CSQ was constructed for this study using the translation of items from the Swedish version of the original CSQ by Jensen and Linton [52].

### Statistical Analysis

Data in this study were part of a larger collection of data and the power calculation to detect a medium effect size difference of the MMR+WEB and MMR group was performed on the work ability index [53] because it was the primary outcome variable for the entire research project. A 5% significance level and 80% power indicated that 64 participants in each intervention group were needed. Considering the possibility of a 20% dropout rate, a sample size of 84 participants in each group was determined to be sufficient.

There were some missing values and cases in the data collection. Isolated missing values in specific questionnaires were imputed according to guidelines for ASES [54] and for GSE [48]. Missing values in CSQ were not imputed. Participants lost to follow-up were handled with intention-to-treat (ITT) analysis, with last observation carried forward (LOCF). Data analysis per protocol were performed and showed nonsignificant differences compared to the analysis of imputed data. The analysis of patient records data was performed with valuable data except for two participants that did not give their consent to follow-up patient records data at 12 months.

Internal consistency for ASES, GSE, and CSQ was tested within our dataset. Excellent internal consistency was found regarding ASES pain (alpha=.9), ASES other symptoms (alpha=.9), and GSE (alpha=.9). Internal consistency for the CSQ subscales were diverting attention (alpha=.6), reinterpreting pain sensations (alpha=.7), catastrophizing (alpha=.7), ignoring sensations (alpha=.6), praying or hoping (alpha=.5), coping self-statements (alpha=.6), and increased behavioral activities (alpha=.3).

Differences in baseline characteristics were tested with independent-samples *t* test, Mann-Whitney *U* test, and chi-square test (Pearson). Repeated measures ANOVA statistics were used to analyze treatment effects between groups over time (time*group), and the whole study population over time (time). Differences between groups in mean changes (delta values) in outcome variables at 4 and 12 months were analyzed with independent-samples *t* test. Because the analysis included several repeated statistical analyses, we choose a more conservative approach of *P*<.01 to be considered as statistically significant instead of *P*<.05.

Effect size was assessed between the MMR+WEB group and the MMR group at the time points 4 and 12 months by calculating Cohen *d* (the mean difference between the groups divided by the pooled standard deviation at baseline). A difference in effect size of 0.2 to 0.5 is regarded as small, between 0.5 and 0.8 as medium, and greater than 0.8 as large [55]. An online calculator was used for this purpose [56].

Data analyses were performed using IBM SPSS version 23 (IBM Corporation).

## Results

### Study Participation

**Figure 1:**
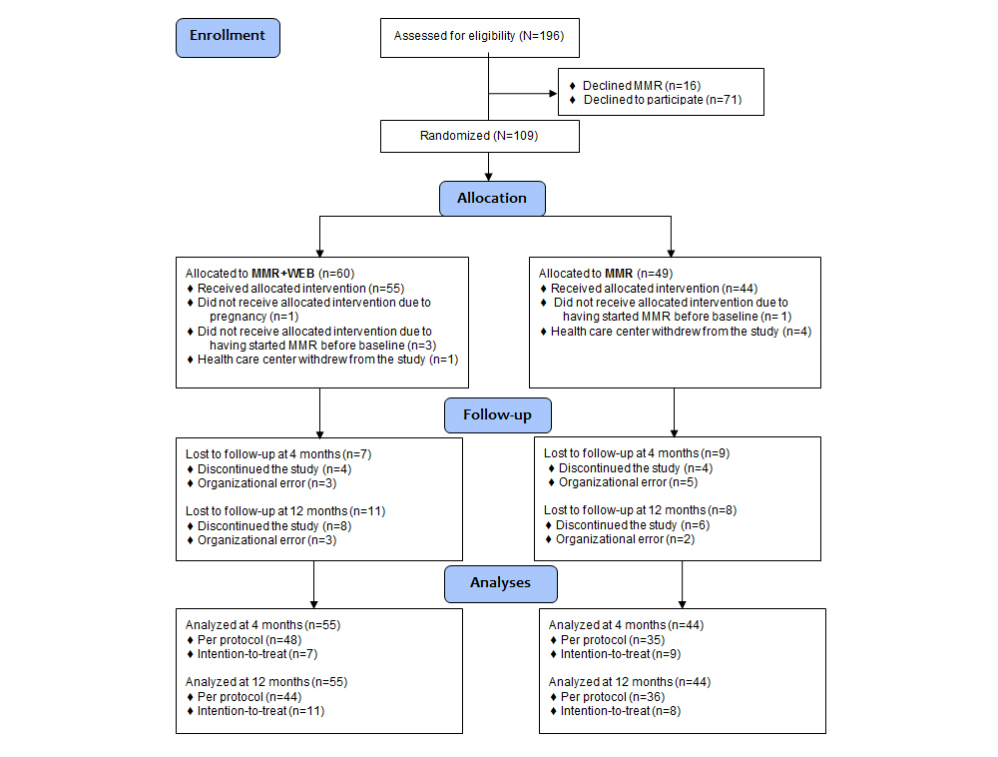
Participant flow diagram. MMR: multimodal rehabilitation; MMR+WEB: multimodal rehabilitation and Web Behavior Change Program for Activity.

The flow of participants through the study is presented in Figure 1. Of the 196 persons assessed for eligibility according to the inclusion and exclusion criteria, 16 (women: n=12, men: n=4; age: mean 46, SD 13 years) declined participation in MMR, with reference to fatigue, time pressure, preferring unimodal treatment, and fear of being stigmatized. In all, 71 persons (49 women and 22 men; mean age 44, SD 12 years) started MMR, but renounced participation in the study due to fatigue, lack of energy, dyslexia, time pressure, wrong timing, or MMR treatment being enough. Other reasons were having no interest or skill with computer work, not being able to work at the computer due to pain, as well as not being interested and motivated to participate in a study.

A total of 109 participants were randomized to MMR+WEB (n=60) or MMR (n=49). However, five participants in each group did not receive MMR and were excluded from the study. At 4 months, 83 of 99 (84%) participants were followed up. Those lost to follow-up were 12 women and four men, aged between 27 and 58 (mean 42, SD 11) years. The follow-up rate at 12 months was 81% (80/99); 13 women and six men, aged between 31 and 63 (mean 44, SD 11) years, were lost to follow-up. Reasons for not being followed up were either participant’s voluntary discontinuation or organizational failure, such as the changing of rehabilitation coordinator or not being able to make contact with the participant. There were no significant differences of baseline characteristics between participants attending follow-up at 12 months and those lost to follow-up.

### Participants’ Characteristics

Participants’ characteristics at baseline are shown in Table 2. Overall, the mean age was 43 (SD 11) years and the majority (84/99) were women. Most participants (81/99) lived with a spouse and approximately 50% (51/99) had children in the household. The education level was higher in the MMR+WEB group with 31% (17/55) of the participants having a university degree compared to 20% (9/44) in the MMR group. More than half (56/99) of the participants in both study groups were working at least 25% at baseline and approximately 75% (76/99) had employment. In the MMR+WEB group, 27% (15/55) of the participants had less than one hour of physical activity per week; in the MMR group, this number was 21% (9/44). The mean body mass index (BMI) was 29 (SD 7) in the MMR+WEB group and 28 (SD 6) in the MMR group, and 20% (20/99) of participants smoked (Table 2).

Overall, participants had pain duration for a mean 78.5 (SD 97.4) months with a mean pain intensity for last 7 days of 65.5 (SD 16.5). The MMR+WEB group showed a significantly higher ÖMPSQ score (mean 136, SD 20) than the MMR group (mean 125, SD 24, *P*=.01). Both study groups showed a mean self-rated overall health state of 46/100 (SD 18) on EuroQol VAS; approximately one-quarter had previous hospital in-patient MMR (Table 2).

**Table 2:** Participants’ characteristics at baseline (N=99) in the multimodal rehabilitation (MMR) and multimodal rehabilitation and Web Behavior Change Program for Activity (MMR+WEB) groups.

Participants’ characteristics		MMR+WEB (n=55)	MMR (n=44)	*P* value
Age (years), mean (SD)		44 (10)	42 (11)	.30
Gender (female), n (%)		47 (86)	37 (84)	.85
Married or cohabitating, n (%)		45 (82)	36 (82)	>.99
Have children in the household, n (%)		28 (51)	23 (52)	.89
**Education level, n (%)**				.17
	Elementary (1-9 years)	8 (14)	10 (23)	
	Secondary education (10-12 years)	30 (55)	25 (57)	
	University (≥13 years)	17 (31)	9 (20)	
**Working condition, n (%)**				
	Permanent or self-employed	40 (73)	28 (64)	
	Temporary employment	5 (9)	3 (7)	
	Unemployed	6 (11)	9 (20)	
	Student	1 (2)	1 (2)	
	Parental leave	0 (0)	0 (0)	
	Outside the labor market	3 (5)	3 (7)	
Working ≥25% of time at baseline		31 (56)	25 (57)	.96
**Physical activity, n (%)**				.47
	<1 hour per week	15 (27)	9 (21)	
	1-3 hours per week	14 (26)	11 (26)	
	>3 hours per week	26 (47)	23 (53)	
Body mass index in kg/m^2^, mean (SD)		29 (7)	28 (6)	.20
Smoking, n (%)		11 (20)	9 (20)	.96
Pain duration in months, mean (SD)		79 (97)	78 (99)	.96
Pain intensity last 7 days (VAS),^a^ mean (SD)		66 (17)	65 (16)	.67
ÖMPSQ,^b^ mean (SD)		136 (20)	125 (24)	.01
EuroQol VAS,^c^ mean (SD)		45 (18)	47^d^ (18)	.54
Previous MMR,^e^ n (%)		14 (26)	10 (23)	.82

^a^ VAS: Visual Analog Scale. Score between zero (no pain) and 100 (worst imaginable pain).

^b^ ÖMPSQ: Örebro Musculoskeletal Pain Screening Questionnaire. Maximum score=210. A score ≥90 indicates a moderate estimated risk for persistent pain and future disability; ≥105 indicates a higher estimated risk.

^c^ Score between zero (worst imaginable health state) and 100 (best imaginable health state).

^d^ n=41.

^e^ History of hospital in-patient multimodal pain rehabilitation.

**Table 3:** Adherence to the Web Behavior Change Program for Activity.

Module	Time spent in module (min)		Users per module,^a^ n (%)
	Mean (SD)	Range	
1	79 (67)	0-345	54 (98)
2	52 (62)	0-259	43 (78)
3	50 (66)	0-377	41 (74)
4	44 (55)	0-179	37 (67)
5	29 (36)	0-158	32 (58)
6	22 (37)	0-167	27 (49)
7	14 (23)	0-79	25 (46)
8	14 (37)	0-215	20 (36)
Total time	304 (267)	0-1142	

^a^ The number of participants that opened the module at some point.

**Table 4:** Feasibility and treatment satisfaction of the Web Behavior Change Program for Activity (Web-BCPA) for the multimodal rehabilitation and BCPA (BCPA+WEB) group (n=55).

Item	Mean (SD)	n
It was easy to use the program	82 (22)	44
It was easy to log in to the program^a^	90 (23)	44
Except for the first introduction, I have self-guided the program^a^	86 (29)	44
It was easy to comprehend the program^a^	90 (17)	44
The graphical design was...^b^	84 (21)	44
The texts have been of good use^a^	84 (24)	44
The assignments have been of good use^a^	73 (27)	42
The videos have been of good use^a^	68 (27)	41
Satisfied with my own efforts in the Web-based program^a^	62 (32)	43
Satisfied with the administrative support in the Web-based program^a,c^	93 (18)	42
I could recommend the Web-based program to others in similar situations to mine^a^	88 (24)	43

^a^ Score ranging from zero (disagree) to 100 (totally agree).

^b^ Score ranging from zero (not at all appealing) to 100 (appealing).

^c^ Support given by the rehabilitation coordinator.

**Table 5:** Satisfaction with multimodal rehabilitation at 4 and 12 months for the multimodal rehabilitation and Web Behavior Change Program for Activity (MMR+WEB) (n=55) and the MMR (n=44) groups.

Item^a^	MMR+WEB		MMR		*P* value
	Mean (SD)	n	Mean (SD)	n	
Satisfied with my multimodal rehabilitation at 4 months	85 (19)	46	65 (25)	35	<.001
Satisfied with own efforts in my multimodal rehabilitation at 4 months	73 (26)	46	66 (26)	35	.20
Satisfied with my multimodal rehabilitation at 12 months	82 (24)	50	66 (28)	39	.003
Satisfied with own efforts in my multimodal rehabilitation at 12 months	74 (25)	50	67 (24)	39	.19

^a^ Score ranging from zero (disagree) to 100 (totally agree).

### Multimodal Rehabilitation Treatment

The multimodal rehabilitation consisted of a mean 30 (SD 8) treatment sessions in the MMR+WEB group and mean 26 (SD 6) in the MMR group. In the MMR+WEB group, 96% (53/55) of the participants had physiotherapy treatment; in the MMR group, it was 95% (42/44). Occupational therapy was attended by 93% (51/55) of the participants in the MMR+WEB group compared to 86% (38/44) in the MMR group. Overall, 78% (43/55) of participants in the MMR+WEB group and 80% (35/44) in the MMR group were treated with psychosocial counseling. In the MMR+WEB group, 96% (53/55) of the participants had treatments by a physician compared to 98% (43/44) in the MMR group; 7% of participants in both the MMR+WEB group (4/55) and the MMR group (3/44) were treated by nurse. The number of team conference meetings were a mean 3 (SD 1) for the MMR+WEB group and mean 2 (SD 1) for the MMR group. In both study groups, 75% (74/99) of all treatments were given during the first 4 months of rehabilitation. At 4 months, 60% (33/55) of the participants in the MMR+WEB group and 70% (31/44) in the MMR group had completed the MMR. At 12 months, the percentage of participants that had completed their rehabilitation was 91% (50/55) in the MMR+WEB group and 95% (42/44) in the MMR group.

### Web Behavior Change Program for Activity Adherence

The mean time spent in the Web-BCPA for all eight modules was 304 minutes (SD 267) or approximately 5 hours. The mean number of modules opened was 5.1 (SD 2.9). A total of 20 of 55 (36%) persons opened all eight modules in the program. The number of users, as well as time spent, decreased with each module. In module 1, mean time spent was 79 (SD 67) minutes, whereas in module 8 the mean time was only 14 (SD 37) minutes. One participant did not open any module (Table 3).

### Web Behavior Change Program for Activity Feasibility and Treatment Satisfaction

Participants rated easiness to comprehend and to log in to the Web-BCPA 90/100. Easiness to use the program and guiding themselves in the program, as well as the graphical design of the Web-BCPA and the applicability of the texts, were rated between 82/100 to 86/100. The lowest mean score was found on the applicability of the exercises and videos (Table 4).

Participants assessed satisfaction with the administrative support in the Web-BCPA from the rehabilitation coordinator as 93/100 and that the Web-based intervention could be recommended to others in similar situation was rated 88/100. Satisfaction with own efforts in the Web-BCPA had the lowest rating (Table 4).

Satisfaction with the MMR was rated significantly higher in the MMR+WEB group at 4 months (*P*<.001) and 12 months (*P*=.003) than in the MMR group. There were no significant differences between the groups at 4 or 12 months regarding participants’ satisfaction with their own efforts in the MMR (Table 5).

### Pain Intensity

Descriptive statistics of mean, minimum, and maximum pain in last 7 days are presented in Table 6. There were no significant differences between groups at baseline for pain variables; however, ratings in the MMR+WEB group tended to be somewhat higher (*P* values not shown). There were no treatment effects between the intervention groups over time (time*group) for mean pain (*P*=.52), minimum pain (*P*=.27), or maximum pain (*P*=.55). There were also not any significant between-group differences in mean changes at the time points 4 and 12 months for pain intensity (Table 6).

**Table 6:** Effects of multimodal rehabilitation and Web Behavior Change Program for Activity (MMR+WEB) on pain intensity as measured with the Visual Analog Scale (VAS) at baseline, 4 months, and 12 months, and mean differences between intervention groups with effect sizes (Cohen *d*).

Outcome measures		MMR+WEB (n=55)	MMR (n=43)	*P* value		Difference MMR+WEB–MMR		Effect size (*d*)
		Mean (SD)	Mean (SD)	Time*group	Time	Mean (95% CI)	*P* value	
**VAS mean^a^**				.52	<.001			
	Baseline	66.1 (16.7)	64.7 (16.2)					
	4 months	59.6 (21.0)	54.8 (21.9)			3.4 (–10.2 to 3.4)	.32	–0.22
	12 months	57.9 (21.8)	56.9 (22.0)			–0.4 (–7.2 to 7.9)	.92	0.02
**VAS minimum^a^**				.27	.47			
	Baseline	42.1 (24.3)	32.8 (23.8)					
	4 months	41.5 (25.6)	29.1 (23.7)			3.1 (–10.5 to 4.3)	.40	–0.13
	12 months	40.3 (26.6)	34.3 (24.9)			–3.2 (–4.9 to 11.3)	.43	0.14
**VAS maximum^a^**				.55	.002			
	Baseline	82.5 (13.5)	79.7 (18.1)					
	4 months	75.8 (19.2)	73.8 (21.3)			–0.8 (–6.4 to 8.0)	.83	0.05
	12 months	75.5 (17.2)	76.5 (18.8)			–3.9 (–3.9 to 11.6)	.32	0.24

^a^ Pain intensity in last 7 days; zero=no pain or discomfort, 100=unbearable pain or discomfort.

**Table 7:** Effects of ultimodal rehabilitation and Web Behavior Change Program for Activity (MMR+WEB) on self-efficacy as measured with the Arthritis Self-Efficacy Scale (ASES) and the General Self-Efficacy Scale (GSE) at baseline, 4 months, and 12 months, and mean differences between intervention groups with effect sizes (Cohen *d*).

Outcome measures		MMR+WEB (n=55)	MMR (n=44)	*P* value		Difference MMR+WEB–MMR		Effect size (*d*)
		Mean (SD)	Mean (SD)	Time*group	Time	Mean (95% CI)	*P* value	
**ASES pain**				.04	.28			
	Baseline	45.8 (21.6)	49.0 (20.4)					
	4 months	50.0 (23.4)	49.3 (21.9)			3.9 (–2.5 to 10.3)	.23	0.19
	12 months	53.2 (22.3)	46.9 (22.2)			9.5 (1.2 to 17.7)	.02	0.45
**ASES other symptoms**				.89	.01			
	Baseline	52.6 (19.2)	52.0 (16.7)					
	4 months	58.1 (21.5)	56.1 (19.8)			1.4 (–4.7 to 7.5)	.65	0.08
	12 months	57.5 (20.5)	55.8 (21.8)			1.2 (–6.7 to 9.0)	.78	0.06
**GSE^a^**				.30	.12			
	Baseline	2.90 (0.60)	2.97 (0.46)					
	4 months	2.88 (0.58)	3.06 (0.53)			–0.10 (–0.22 to 0.02)	.11	–0.10
	12 months	2.93 (0.62)	3.08 (0.56)			–0.07 (–0.22 to 0.07)	.33	–0.15

^a^ MMR+WEB group (n=54) and MMR group (n=43).

**Table 8:** Effects of multimodal rehabilitation and Web Behavior Change Program for Activity (MMR+WEB) on coping as measured with the two-item Coping Strategies Questionnaire (CSQ) at baseline, 4 months, and 12 months, and mean differences between intervention groups with effect sizes (Cohen *d*).

CSQ subscales		MMR+WEB (n=54)	MMR (n=44)	*P* value		Difference MMR+WEB–MMR		Effect size (*d*)
		Mean (SD)	Mean (SD)	Time*group	Time	Mean (95% CI)	*P* value	
**Diverting attention^a^**				.61	.14			
	Baseline	2.9 (1.4)	2.8 (1.5)					
	4 months	3.2 (1.4)	2.9 (1.7)			0.2 (–0.2 to 0.6)	.36	0.14
	12 months	3.1 (1.5)	3.0 (1.7)			–0.0 (–0.6 to 0.5)	.92	–0.00
**Reinterpreting pain sensations^a,b^**				.63	.12			
	Baseline	1.8 (1.4)	1.7 (1.4)					
	4 months	2.1 (1.3)	1.8 (1.4)			0.2 (–0.3 to 0.6)	.46	0.14
	12 months	2.1 (1.4)	2.0 (1.4)			–0.0 (–0.6 to 0.6)	.98	–0.00
**Catastrophizing**				.003	.002			
	Baseline	3.2 (1.4)	2.8 (1.2)					
	4 months	2.8 (1.4)	2.8 (1.4)			–0.4 (–0.9 to 0.0)	.06	0.31
	12 months	2.4 (1.4)	2.8 (1.4)			–0.8 (–0.3 to –1.3)	.001	0.61
**Ignoring sensations^a^**				.03	.30			
	Baseline	2.7 (1.2)	2.8 (1.2)					
	4 months	2.9 (1.1)	2.9 (1.3)			0.1 (–0.3 to 0.5)	.06	0.08
	12 months	3.0 (1.3)	2.5 (1.3)			0.6 (0.1 to 1.0)	.02	0.50
**Praying or hoping**				.78	.33			
	Baseline	2.7 (1.6)	2.6 (1.5)					
	4 months	2.8 (1.6)	2.5 (1.7)			0.2 (–0.3 to 0.6)	.52	0.13
	12 months	2.6 (1.6)	2.4 (1.5)			0.1 (–0.4 to 0.6)	.77	0.06
**Coping self-statements**				.48	.42			
	Baseline	3.1 (1.1)	3.1 (1.3)					
	4 months	3.0 (1.2)	2.9 (1.3)			0.0 (–0.4 to 0.4)	.93	0.25
	12 months	3.2 (1.3)	2.9 (1.4)			0.2 (–0.2 to 0.7)	.32	0.13
**Increased behavioral activities^a^**				.10	.15			
	Baseline	3.3 (1.1)	3.3 (1.2)					
	4 months	3.4 (1.0)	3.1 (1.3)			0.4 (0.00 to 0.8)	.047	0.26
	12 months	3.5 (1.0)	3.4 (1.4)			0.2 (–0.2 to 0.1)	.39	0.09

^a^ MMR group (n=43).

^b^ MMR+WEB group (n=53).

An overall significant improvement over time (time) was found in the whole study group for mean (*P*<.001) and maximum pain (*P*=.002) (Table 6).

### Self-Efficacy

Table 7 shows the descriptive statistics for ASES pain, ASES other symptoms, and GSE. There were no significant differences between groups at baseline for variables of self-efficacy (*P* values not shown). There were no treatment effects over time (time*group) between the MMR+WEB group and the MMR group for ASES pain (*P*=.04), ASES other symptoms (*P*=.89), and GSE (*P*=.30). There were also not any between-group differences in mean changes at the time points 4 and 12 months for ASES pain, ASES other symptoms, and GSE (Table 7).

There were no improvements over time (time) for the whole study group regarding ASES pain (*P*=.28), ASES other symptoms (*P*=.01), and GSE (*P*=.12).

### Coping

Descriptive statistics for the seven subscales of the two-item CSQ is presented in Table 8. There were no significant differences between groups at baseline for CSQ subscales (*P* values not shown). The catastrophizing subscale demonstrated significant treatment effects between groups over time (time*group; *P*=.003) in favor of the MMR+WEB group. The differences between the groups in mean changes were not significant at time point 4 months (*P*=.06, *d*=0.31), whereas they were significant at 12 months (*P*=.001) with a medium to large effect size (*d=* 0.61). There were no treatment effects between the groups over time (time*group) for diverting attention, reinterpreting pain sensations, ignoring sensations, praying or hoping, coping self-statements, and increased behavioral activities subscales (Table 8).

Treatment effects over time (time) for the whole study group was found regarding catastrophizing (*P*=.002). There were no significant improvements over time for the whole study group regarding diverting attention, reinterpreting pain sensations, ignoring sensations, praying or hoping, coping self-statements, and increased behavioral activities subscales (Table 8).

## Discussion

### Principal Findings

This RCT studied the effects of the self-guided Web-BCPA in combination with MMR for participants with persistent musculoskeletal pain in primary health care. Overall, we found decreased catastrophizing in the MMR+WEB group compared to the MMR group. Previously, both self-guided [39] and therapist-guided [33,34,57] Web-based interventions for chronic pain have reported treatment effects of decreased catastrophizing, The treatment effects of catastrophizing in our study showed an effect size of *d*=0.61. This is higher than the reported Hedge’s *g*=.33 in the systematic review of Web-based interventions for chronic pain by Buhrman et al [5] and is in line with the findings of Dear et al [57] from a therapist-supported Web intervention. There were no treatment effects of any other of the CSQ subscales, which is in line with Buhrman et al [33,34]. This indicates that content and form of delivery does not seem to affect coping strategies except for catastrophizing. With the limit of significance set to *P*<.01, we did not find any treatment effects regarding self-efficacy for pain, self-efficacy for other symptoms, or general self-efficacy. Increased self-efficacy to control pain has been reported for a Web-based intervention for pain management with therapist support [57], but Chiauzzi et al [36] found no treatment effects of self-efficacy from a self-guided Web-based intervention. However, the reduction of catastrophizing indicated that the Web-BCPA content had changed the participants’ negative beliefs about pain. The educational text in the first module of the Web-BCPA explained persistent pain from the physiological and psychological perspective, and most participants may have assimilated this knowledge. In addition, assignments in the Web-BCPA focused on personal goals in life and not on pain experiences, which is supported by earlier research that decreased focusing on pain signals are effective in pain rehabilitation [24,26,29].

Decreased pain intensity has previously been demonstrated from self-guided Web-based interventions for pain management compared with standard care by physician [37,39]. In this study, we found no effects on pain intensity from the Web-BCPA. There were overall effects over time for the whole study group regarding mean and maximum pain intensity in the last 7 days, which indicates that MMR can be an effective intervention to reduce perceived pain. Because this was observed without a placebo control group this should be interpreted cautiously; however, it is in line with the findings from Kamper et al [19] that MMR reduced pain compared to standard treatment. Participants in both study groups had MMR according to national and regional guidelines with the mean number of treatments above the recommended lower limit and 75% of the treatments within the first 4 months of rehabilitation. The majority of the participants were treated with psychosocial counseling in their MMR, which may have included coaching according to cognitive behavioral therapy. The fact that both intervention groups received MMR treatment may have reduced the therapeutic power of the Web-BCPA intervention. There were no overall effects over time for the whole study group regarding any of the self-efficacy scales (although self-efficacy to control other symptoms showed a statistical value close to significant; *P=*.01) or regarding six of seven CSQ subscales.

We found that participants in the MMR+WEB group were more satisfied with their MMR both at 4 and 12 months (mean 82/100, SD 24, *P=*.003) compared to persons in the MMR group (mean 66/100, SD 28). On the other hand, we found no differences regarding satisfaction with own effort in the MMR. The Web-BCPA treatment satisfaction and feasibility were rated good to excellent. Satisfaction with treatment has been found to relate to adherence and compliance to treatment [58,59], and is associated with patients’ perceptions of a positive patient-health care professional relationship [58]. The participants may have perceived a more complete rehabilitation by taking part in both MMR and the Web-BCPA. However, we found that the mean time spent in the Web-BCPA was less than we had expected (approximately 5 hours during a treatment period of 16 weeks). The measure of time spent in Web-based programs is rarely reported in the literature; therefore, there are few references to compare our results with. Lorig et al [60] tested a 6-week Web intervention for patients with persistent pain, with the recommendation to spend 1 to 2 hours each week in the program divided by three log-in occasions. The number of log-ins was measured and, assuming that the participants in the Lorig et al study had followed the recommendations [60], those participants would have spent at least 10 to 20 hours in their Web-based program. In our study, only nine participants reached that time range in the Web-BCPA. The Web-BCPA adherence decreased with each module, but this do not indicate if participants discontinued the Web-BCPA over time because most assignments and exercises were introduced in the early modules and then repeated in the sequential modules. It is likely that a participant who had started on a behavior change plan or relaxation exercises in one of the first modules returned to the same module to continue their work. The minutes spent in the Web-based program were monitored for each module and not related to week. There may be more appropriate ways to assess adherence in Web-based interventions, such as measuring number of log-ins and clicks in relation to time spent within the module, but this was not an option in our study due to limitations of the program software. However, the lower usage of later modules suggests that many participants did not assimilate all the educational texts and missed information about controlling other symptoms such as fatigue, stress, and sleep disturbance. Another limitation in this study is that we did not assess aspects of cognitive activity in the Web-BCPA, such as the acquiring of skills and knowledge, goal setting, and solving problems. Ruehlman et al [39] assessed pain knowledge (topics addressed within the Web-based intervention) and found improvements among participants in a Web-based intervention compared with treatment as usual.

### Strengths and Limitations

The strengths in our study are the RCT design and that the Web-BCPA was implemented in a MMR context in primary health care, which to our knowledge is the first reported in the field. However, the number of participants in the analysis reached 77% of the calculated number needed, thus the study is underpowered to detect small improvements in outcome variables and increases the risk of type II errors. Because the dropout rate at 12 months was modest (18%) and we used ITT analysis, our findings may be less prone to bias. But all missing data mean uncertainty and reduced reliability and interpretability of the results. In this study, we had an ITT approach and used the LOCF method for imputation of data. LOCF has limitations, but handles data in a conservative way by assuming no treatment effects over time, which reduces the risk of overestimating of results. Because LOCF underestimates variance, it is possible that methods such as multiple imputation would generate more appropriate results. For exploratory reasons, we also performed per protocol analyses, which generated similar results as the LOCF analyses. We also decided to be more conservative with a significance level of *P*<.01 due to the number of variables in our data collection to minimize the risk of overestimating results.

The Web-BCPA was redesigned with alterations made to fit participants with persistent pain in an early stage, with less developed chronicity [12]. We believed that being in an early stage of persistent pain would entail better physical and psychosocial resources to self-guide in the Web-based program and to assimilate the content. However, partly because of organizational factors at the health care centers, the participants in our study suffered from longer pain duration (approximately 6.5 years) and higher levels of pain than we anticipated when designing the study. The levels of pain intensity were higher than was previously reported in other MMR interventions [61-63]. Together with the participants’ high ÖMPSQ scores, this may indicate symptoms of exhaustion and stress among the participants [12], symptoms that have been found to reduce participation in Web-based interventions [3]. This may be a possible explanation for the low adherence of the Web-BCPA, together with a probably variable motivation level of participants randomized to the Web-BCPA. Most earlier studies using Web-based intervention have used a voluntary application for inclusion, whereas in our study participants searched health care for pain management and could end up with the Web-BCPA. This nonvoluntary randomization to Web-BCPA might partly explain the low adherence. Also, it is possible that the Web-BCPA content was extensive and may have been difficult for this group of patients to take on. Time and motivation are reported reasons for not using Web-based treatments [64], and patients with pain problems may prefer face-to-face therapy when there is a choice [64,65]. Our experience was that persons accepted treatment with MMR but declined participation in the study. The proportion of men that started MMR but declined participation in the study was higher (30%) than the percentage of men included in the study (15%). In addition, they were of lower age both compared to nonparticipant women and the total study population. Similar characteristics (male gender and young age) have previously been found to be predictors of not completing Web-based interventions [3], and Web-based interventions have suffered from high dropout rates, also with optional participation [3,65]. In this study, the Web-BCPA was self-guided, which may also have affected adherence. The participants may have needed more professional support, such as an extended introduction and/or counseling in the Web-BCPA content, to find the program valuable.

The two-item CSQ was used to assess the participant’s coping strategies and, to our knowledge, this is the first time it was tested on a Swedish population. The internal consistency of the catastrophizing and reinterpreting pain sensations subscales was acceptable (alpha=.7), but the other five subscales did not have a satisfying Cronbach alpha. Considering this, our results must be regarded with caution. The two-item CSQ needs to be further tested for reliability and validity.

### Conclusion

In this study, the self-guided Web-BCPA was added to MMR. There were no treatment effects regarding self-efficacy, perceived pain intensity, or most coping strategies in this study group of persons with long-lasting pain conditions. However, participants treated with MMR in combination with the Web-BCPA reduced their catastrophic thinking compared to participants in MMR. In addition, they were more satisfied with their MMR. The Web-BCPA adherence was low and may have been influenced by participants’ baseline characteristics and their symptom panorama. It may be important to consider the individual’s motivation and ability when suggesting a Web-based intervention. Adding counseling to the Web-BCPA might increase adherence and the use of the Web-based intervention.

## Appendix

Multimedia Appendix 1 Screenshot of the web-BCPA.

Multimedia Appendix 2 Video file of the web-BCPA.

## Abbreviations

ASES: Arthritis Self-Efficacy Scale
BMI: body mass index
CSQ: Coping Strategies Questionnaire
GSE: General Self-Efficacy Scale
ITT: intention-to-treat
LOCF: last observation carried forward
MMR: multimodal rehabilitation
MMR+WEB: multimodal rehabilitation and Web-BCPA
ÖMPSQ: Örebro Musculoskeletal Pain Screening Questionnaire
RCT: randomized controlled trial
VAS: Visual Analog Scale
Web-BCPA: Web Behavior Change Program for Activity
