# Supplementary figures and images for: Effects of the Web Behavior Change Program for Activity and Multimodal Pain Rehabilitation: Randomized Controlled Trial
Source: J Med Internet Res. 2016 Oct 5;18(10):e265. doi: 10.2196/jmir.5634 (PMC5071618; doi:10.2196/jmir.5634)

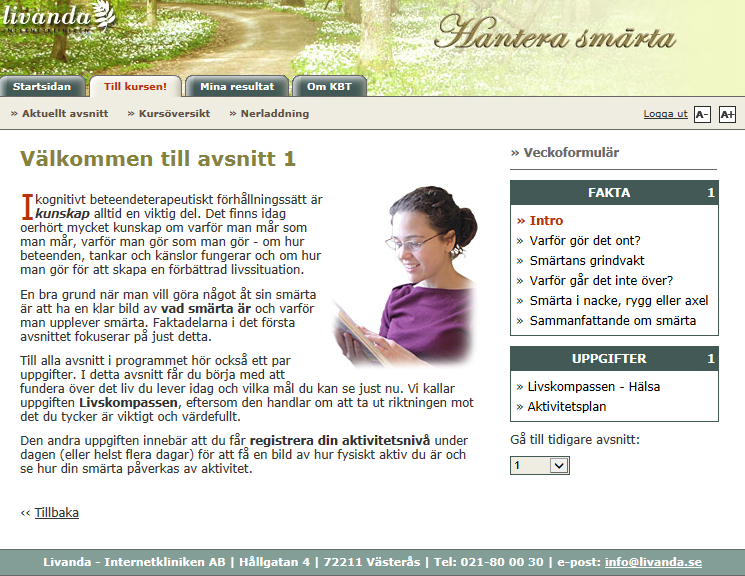

Supplement: Multimedia Appendix 1 [file jmir_v18i10e265_app1.png]
